# The genome sequence of the Tipped Oak Case-bearer, *Coleophora flavipennella *(Duponchel 1843)

**DOI:** 10.12688/wellcomeopenres.19917.1

**Published:** 2024-01-03

**Authors:** Douglas Boyes, Mark L. Blaxter, University of Oxford and Wytham Woods Genome Acquisition Lab, Darwin Tree of Life Barcoding collective, Wellcome Sanger Institute Tree of Life programme, Wellcome Sanger Institute Scientific Operations: DNA Pipelines collective, Tree of Life Core Informatics collective, Darwin Tree of Life Consortium

**Affiliations:** 1 UK Centre for Ecology & Hydrology, Wallingford, England, UK; 2 Wellcome Sanger Institute, Hinxton, England, UK

**Keywords:** Coleophora flavipennella, Tipped Oak Case-bearer, genome sequence, chromosomal, Lepidoptera

## Abstract

We present a genome assembly from an individual female *Coleophora flavipennella* (the Tipped Oak Case-bearer; Arthropoda; Insecta; Lepidoptera; Coleophoridae). The genome sequence is 989.3 megabases in span. Most of the assembly is scaffolded into 57 chromosomal pseudomolecules, including the W and Z sex chromosomes. The mitochondrial genome has also been assembled and is 15.77 kilobases in length.

## Species taxonomy

Eukaryota; Metazoa; Eumetazoa; Bilateria; Protostomia; Ecdysozoa; Panarthropoda; Arthropoda; Mandibulata; Pancrustacea; Hexapoda; Insecta; Dicondylia; Pterygota; Neoptera; Endopterygota; Amphiesmenoptera; Lepidoptera; Glossata; Neolepidoptera; Heteroneura; Ditrysia; Gelechioidea; Coleophoridae; *Coleophora*; *Coleophora flavipennella* (Duponchel, 1843) (NCBI:txid1100951).

## Background

Moth larvae are important herbivores in agricultural and natural ecosystems, and in turn act as food for many insectivores. The close coordination of insectivorous bird breeding with spring budburst is linked through the hatching of larvae from a wide variety of insects, especially moth species, that feast on newly emerged leaves. This general phenomenon has been analysed at a very fine scale through long term studies of both tree bud burst phenology ( Cole & Sheldon, 2017) and nesting timing of marked birds (great tits, *Parus major*) ( Hinks *et al.*, 2015) at Wytham Woods, near Oxford.

Larval moths have a variety of associations with their host plants, and many have evolved mechanisms to protect themselves from predation. One such group is the “case bearing” micromoths of the family Coleophoridae (see Bauer *et al.* (2012)), in which the larvae form a case from mined leaf fragments, attached and strengthened using silk. As part of our wider goal to generate genomic resources for the British and Irish biota ( Blaxter *et al.*, 2022), we present the chromosomally-complete genome sequence of one such case bearing coleophorid moth, *Coleophora flavipennella*, isolated from Wytham Woods. Larval *C. flavipennella* feed on broadleaved trees, including *Quercus robur* and *Quercus petraea*, which are abundant in Wytham Woods. While not particularly commonly recorded, in Britain and Ireland *C. flavipennella* is found south of a line from the Wash in East Anglia to the Lake District ( https://species.nbnatlas.org/species/NHMSYS0021142811; for Irish records see, for example, ( Bond, 1996). *C. flavipennella* is not thought to be especially endangered ( Davis, 2012), but is likely to be being impacted by anthropogenic disturbance and climate change, especially as these affect the phenology of its host plants.

## Genome sequence report

**Figure 1.**
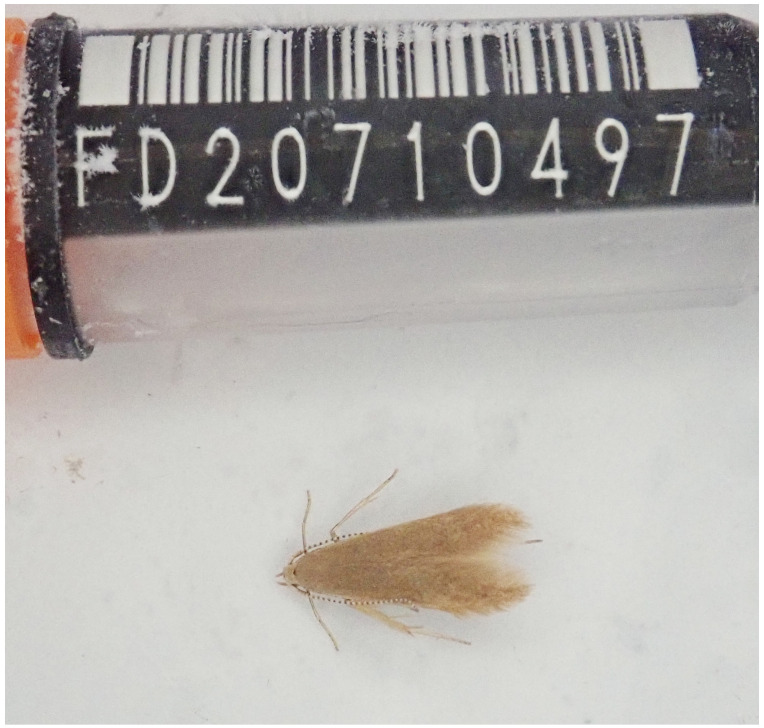
Photograph of the *Coleophora flavipennella* (ilColFlav1) specimen used for genome sequencing.

The genome was sequenced from one female *Coleophora flavipennella* ( Figure 1) collected from Wytham Woods, Oxfordshire, UK (51.77, –1.34). A total of 23-fold coverage in Pacific Biosciences single-molecule HiFi long reads was generated. Primary assembly contigs were scaffolded with chromosome conformation Hi-C data. Manual assembly curation corrected 63 missing joins or mis-joins and removed 15 haplotypic duplications, reducing the assembly length by 0.24% and the scaffold number by 11.9%, and increasing the scaffold N50 by 1.59%.

**Figure 2.**
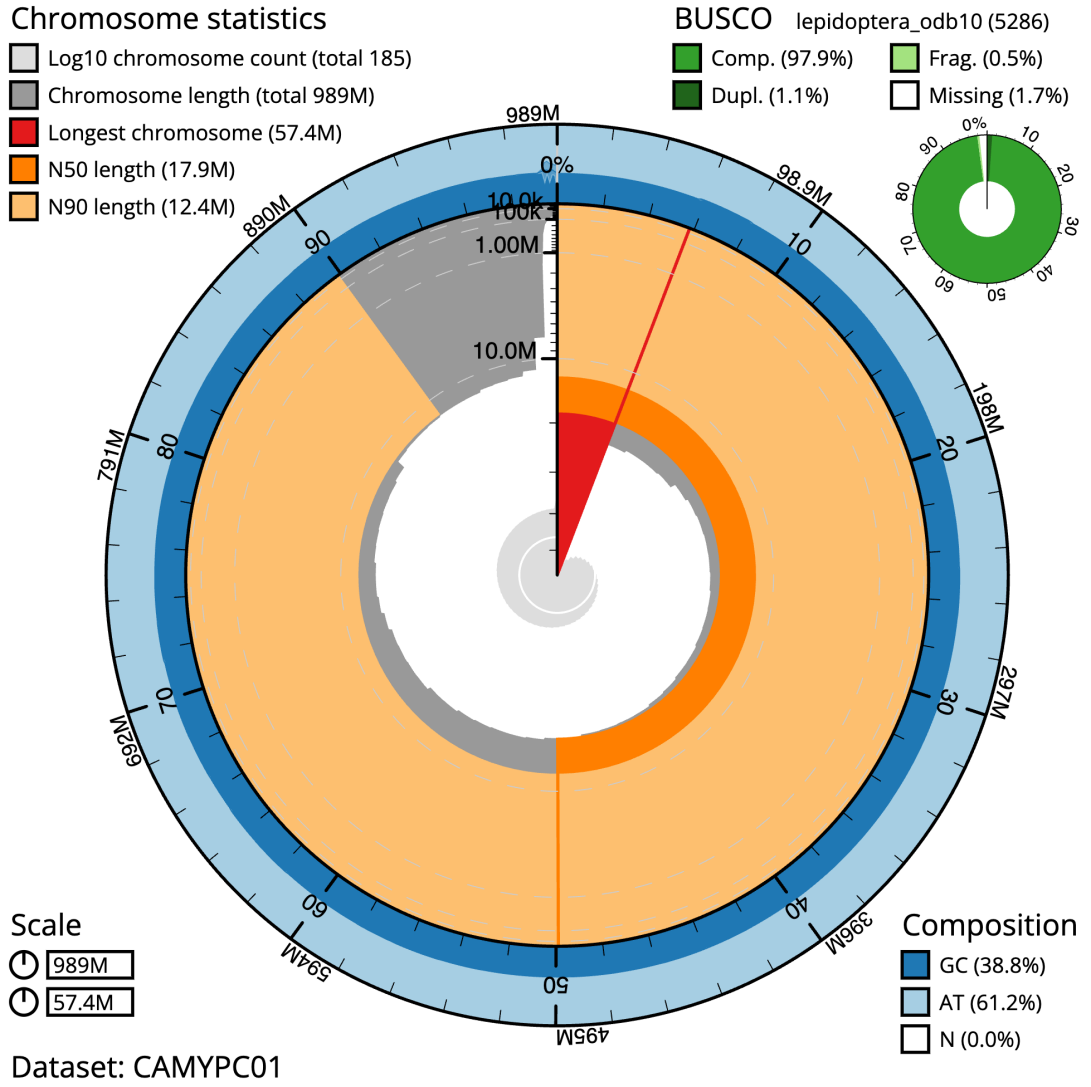
Genome assembly of *Coleophora flavipennella*, ilColFlav1.1: metrics. The BlobToolKit Snailplot shows N50 metrics and BUSCO gene completeness. The main plot is divided into 1,000 size-ordered bins around the circumference with each bin representing 0.1% of the 989,272,584 bp assembly. The distribution of scaffold lengths is shown in dark grey with the plot radius scaled to the longest scaffold present in the assembly (57,400,957 bp, shown in red). Orange and pale-orange arcs show the N50 and N90 scaffold lengths (17,852,142 and 12,400,742 bp), respectively. The pale grey spiral shows the cumulative scaffold count on a log scale with white scale lines showing successive orders of magnitude. The blue and pale-blue area around the outside of the plot shows the distribution of GC, AT and N percentages in the same bins as the inner plot. A summary of complete, fragmented, duplicated and missing BUSCO genes in the lepidoptera_odb10 set is shown in the top right. An interactive version of this figure is available at https://blobtoolkit.genomehubs.org/view/ilColFlav1.1/dataset/CAMYPC01/snail.

The final assembly has a total length of 989.3 Mb in 184 sequence scaffolds with a scaffold N50 of 17.9 Mb ( Table 1). Most (99.38%) of the assembly sequence was assigned to 57 chromosomal-level scaffolds, representing 55 autosomes and the W and Z sex chromosomes. Chromosome-scale scaffolds confirmed by the Hi-C data are named in order of size ( Figure 2– Figure 5; Table 2). While not fully phased, the assembly deposited is of one haplotype. Contigs corresponding to the second haplotype have also been deposited. The mitochondrial genome was also assembled and can be found as a contig within the multifasta file of the genome submission.

**Table 1.** Genome data for *Coleophora flavipennella*, ilColFlav1.1.

Project accession data		
Assembly identifier	ilColFlav1.1	
Species	*Coleophora flavipennella*	
Specimen	ilColFlav1	
NCBI taxonomy ID	1100951	
BioProject	PRJEB56048	
BioSample ID	SAMEA10978959	
Isolate information	ilColFlav1, female: whole organism (DNA sequencing) ilColFlav2: whole organism (Hi-C data)	
Assembly metrics *		*Benchmark*
Consensus quality (QV)	62.1	*≥ 50*
*k*-mer completeness	100%	*≥ 95%*
BUSCO **	C:97.9%[S:96.8%,D:1.1%],F:0.5%,M:1.7%,n:5,286	*C ≥ 95%*
Percentage of assembly mapped to chromosomes	99.38%	*≥ 95%*
Sex chromosomes	Z and W chromosomes	*localised homologous pairs*
Organelles	Mitochondrial genome assembled	*complete single alleles*
Raw data accessions		
PacificBiosciences SEQUEL II	ERR10224922	
Hi-C Illumina	ERR10297813	
Genome assembly		
Assembly accession	GCA_947284805.1	
*Accession of alternate haplotype*	GCA_947285645.1	
Span (Mb)	989.3	
Number of contigs	615	
Contig N50 length (Mb)	4.0	
Number of scaffolds	184	
Scaffold N50 length (Mb)	17.9	
Longest scaffold (Mb)	57.4	

* Assembly metric benchmarks are adapted from column VGP-2020 of “Table 1: Proposed standards and metrics for defining genome assembly quality” from ( Rhie *et al.*, 2021). ** BUSCO scores based on the lepidoptera_odb10 BUSCO set using v5.3.2. C = complete [S = single copy, D = duplicated], F = fragmented, M = missing, n = number of orthologues in comparison. A full set of BUSCO scores is available at https://blobtoolkit.genomehubs.org/view/ilColFlav1.1/dataset/CAMYPC01/busco.

**Figure 3.**
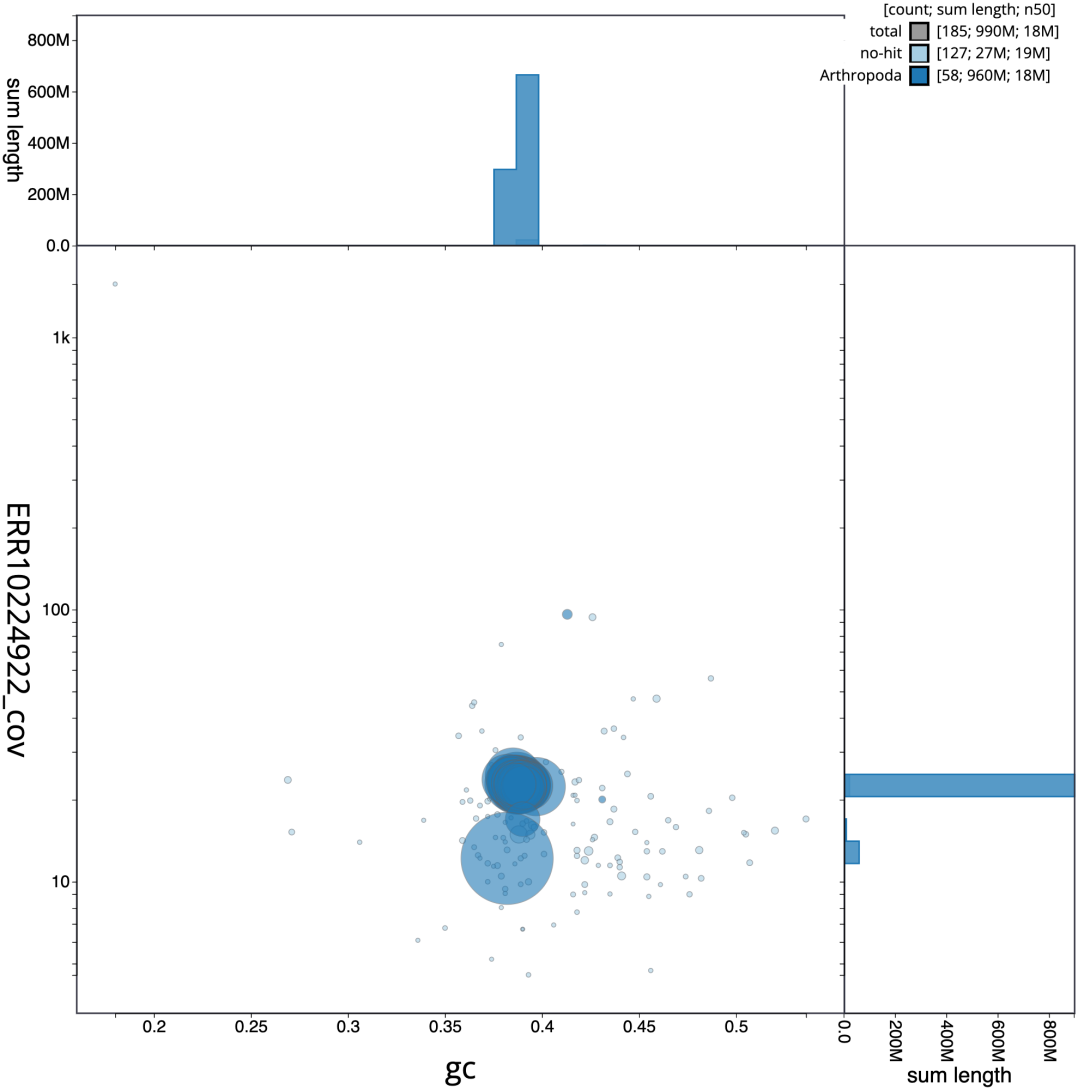
Genome assembly of *Coleophora flavipennella*, ilColFlav1.1: BlobToolKit GC-coverage plot. Scaffolds are coloured by phylum. Circles are sized in proportion to scaffold length. Histograms show the distribution of scaffold length sum along each axis. An interactive version of this figure is available at https://blobtoolkit.genomehubs.org/view/ilColFlav1.1/dataset/CAMYPC01/blob.

**Figure 4.**
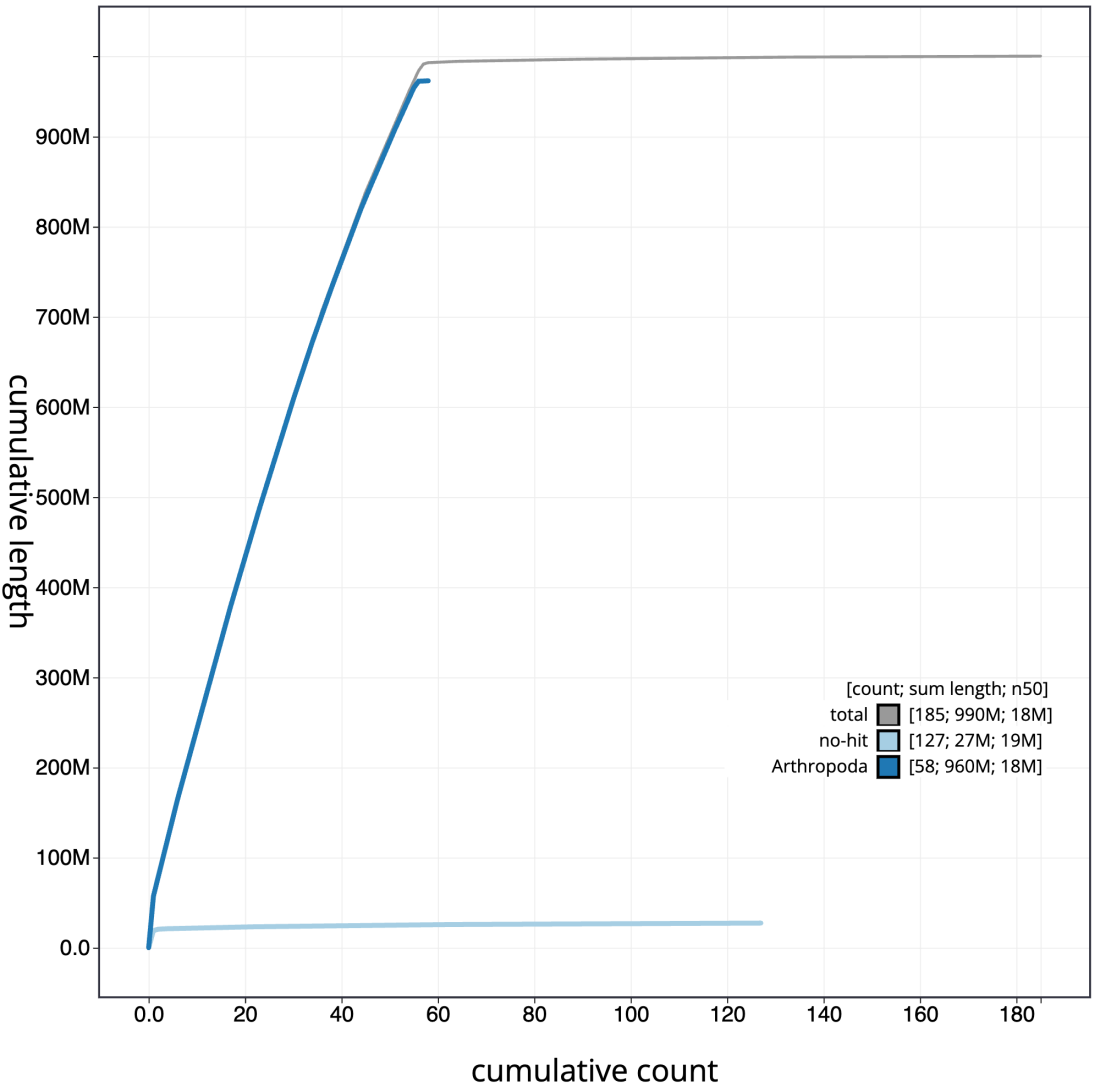
Genome assembly of *Coleophora flavipennella*, ilColFlav1.1: BlobToolKit cumulative sequence plot. The grey line shows cumulative length for all scaffolds. Coloured lines show cumulative lengths of scaffolds assigned to each phylum using the buscogenes taxrule. An interactive version of this figure is available at https://blobtoolkit.genomehubs.org/view/ilColFlav1.1/dataset/CAMYPC01/cumulative.

**Figure 5.**
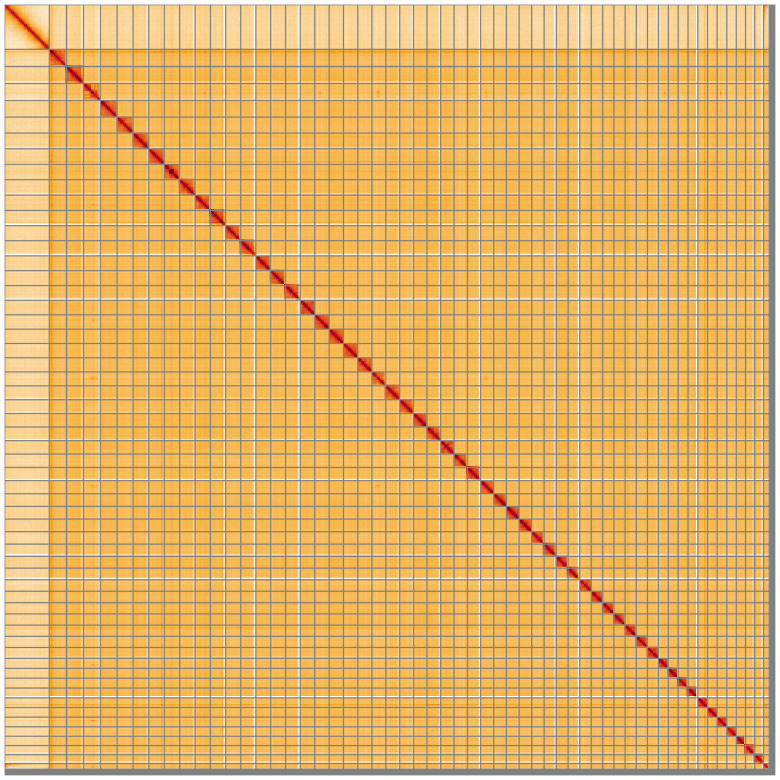
Genome assembly of *Coleophora flavipennella*, ilColFlav1.1: Hi-C contact map of the ilColFlav1.1 assembly, visualised using HiGlass. Chromosomes are shown in order of size from left to right and top to bottom. An interactive version of this figure may be viewed at https://genome-note-higlass.tol.sanger.ac.uk/l/?d=NQR3kVCgS8K4sm8_AoCEWA.

**Table 2.** Chromosomal pseudomolecules in the genome assembly of *Coleophora flavipennella*, ilColFlav1.

INSDC accession	Chromosome	Length (Mb)	GC%
OX369253.1	1	22.14	39.5
OX369254.1	2	21.68	38.5
OX369255.1	3	21.66	38.5
OX369256.1	4	21.45	39.0
OX369257.1	5	20.5	38.5
OX369258.1	6	19.75	38.5
OX369259.1	7	20.07	39.0
OX369260.1	8	19.9	39.0
OX369261.1	9	19.8	38.5
OX369262.1	10	19.66	39.0
OX369263.1	11	19.45	38.5
OX369264.1	12	19.39	39.0
OX369265.1	13	19.32	38.5
OX369266.1	14	19.11	38.5
OX369267.1	15	19.07	39.0
OX369268.1	16	18.83	39.0
OX369269.1	17	18.67	38.5
OX369270.1	18	18.39	38.5
OX369271.1	19	18.35	38.5
OX369272.1	20	18.26	39.0
OX369273.1	21	18.19	38.5
OX369274.1	22	17.86	38.5
OX369275.1	23	17.85	38.5
OX369276.1	24	17.57	38.5
OX369277.1	25	17.41	39.0
OX369278.1	26	17.41	39.0
OX369279.1	27	17.29	39.0
OX369280.1	28	17.16	38.5
OX369281.1	29	16.85	38.5
OX369282.1	30	16.81	39.0
OX369283.1	31	16.28	39.0
OX369284.1	32	16.23	38.0
OX369285.1	33	16.14	38.5
OX369286.1	34	16.05	39.0
OX369287.1	35	15.57	38.5
OX369288.1	36	15.11	39.5
OX369289.1	37	14.86	39.0
OX369290.1	38	14.85	39.0
OX369291.1	39	14.57	39.0
OX369292.1	40	14.51	39.0
OX369293.1	41	14.41	39.0
OX369294.1	42	14.31	39.0
OX369295.1	43	14.26	39.0
OX369296.1	44	13.98	38.5
OX369297.1	45	12.76	39.0
OX369298.1	46	12.66	39.0
OX369299.1	47	12.63	38.5
OX369300.1	48	12.56	39.0
OX369301.1	49	12.4	39.0
OX369302.1	50	12.39	39.0
OX369303.1	51	12.35	39.0
OX369304.1	52	12.04	39.0
OX369305.1	53	11.97	39.0
OX369306.1	54	11.84	39.0
OX369307.1	55	11.2	38.5
OX369309.1	W	7.28	39.0
OX369308.1	Z	57.4	38.0
OX369310.1	MT	0.02	18.0

The estimated Quality Value (QV) of the final assembly is 62.1 with *k*-mer completeness of 100%, and the assembly has a BUSCO v5.3.2 completeness of 97.9% (single = 96.8%, duplicated = 1.1%), using the lepidoptera_odb10 reference set ( *n* = 5,286).

Metadata for specimens, spectral estimates, sequencing runs, contaminants and pre-curation assembly statistics can be found at https://links.tol.sanger.ac.uk/species/1100951.

## Methods

### Sample acquisition and nucleic acid extraction

Two *Coleophora flavipennella* specimens were collected from Wytham Woods, Oxfordshire (biological vice-country Berkshire), UK (latitude 51.77, longitude –1.34) on 2021-07-17 using a light trap. The specimens were collected and identified by Douglas Boyes (University of Oxford) and snap-frozen on dry ice. The specimen used for DNA sequencing was ID Ox001692 (ToLID ilColFlav1), while the specimen with ID Ox001694 (ToLID ilColFlav2) was used for Hi-C scaffolding.

DNA was extracted at the Tree of Life laboratory, Wellcome Sanger Institute (WSI). The ilColFlav1 sample was weighed and dissected on dry ice with tissue set aside for Hi-C sequencing. Tissue from the whole organism was disrupted using a Nippi Powermasher fitted with a BioMasher pestle. High molecular weight (HMW) DNA was extracted using the Qiagen MagAttract HMW DNA extraction kit. HMW DNA was sheared into an average fragment size of 12–20 kb in a Megaruptor 3 system with speed setting 30. Sheared DNA was purified by solid-phase reversible immobilisation using AMPure PB beads with a 1.8X ratio of beads to sample to remove the shorter fragments and concentrate the DNA sample. The concentration of the sheared and purified DNA was assessed using a Nanodrop spectrophotometer and Qubit Fluorometer and Qubit dsDNA High Sensitivity Assay kit. Fragment size distribution was evaluated by running the sample on the FemtoPulse system.

### Sequencing

Pacific Biosciences HiFi circular consensus DNA sequencing libraries were constructed according to the manufacturers’ instructions. DNA sequencing was performed by the Scientific Operations core at the WSI on a Pacific Biosciences SEQUEL II (HiFi) instrument. Hi-C data were also generated from whole organism tissue of ilColFlav2 using the Arima2 kit and sequenced on the Illumina NovaSeq 6000 instrument.

### Genome assembly, curation and evaluation

Assembly was carried out with Hifiasm ( Cheng *et al.*, 2021) and haplotypic duplication was identified and removed with purge_dups ( Guan *et al.*, 2020). The assembly was then scaffolded with Hi-C data ( Rao *et al.*, 2014) using YaHS ( Zhou *et al.*, 2023). The assembly was checked for contamination and corrected using the gEVAL system ( Chow *et al.*, 2016) as described previously ( Howe *et al.*, 2021). Manual curation was performed using gEVAL, HiGlass ( Kerpedjiev *et al.*, 2018) and Pretext ( Harry, 2022). The mitochondrial genome was assembled using MitoHiFi ( Uliano-Silva *et al.*, 2023), which runs MitoFinder ( Allio *et al.*, 2020) or MITOS ( Bernt *et al.*, 2013) and uses these annotations to select the final mitochondrial contig and to ensure the general quality of the sequence.

A Hi-C map for the final assembly was produced using bwa-mem2 ( Vasimuddin *et al.*, 2019) in the Cooler file format ( Abdennur & Mirny, 2020). To assess the assembly metrics, the *k*-mer completeness and QV consensus quality values were calculated in Merqury ( Rhie *et al.*, 2020). This work was done using Nextflow ( Di Tommaso *et al.*, 2017) DSL2 pipelines “sanger-tol/readmapping” ( Surana *et al.*, 2023a) and “sanger-tol/genomenote” ( Surana *et al.*, 2023b). The genome was analysed within the BlobToolKit environment ( Challis *et al.*, 2020) and BUSCO scores ( Manni *et al.*, 2021; Simão *et al.*, 2015) were calculated.

Table 3 contains a list of relevant software tool versions and sources.

**Table 3.** Software tools: versions and sources.

Software tool	Version	Source
BlobToolKit	4.0.7	https://github.com/blobtoolkit/blobtoolkit
BUSCO	5.3.2	https://gitlab.com/ezlab/busco
gEVAL	N/A	https://geval.org.uk/
Hifiasm	0.16.1-r375	https://github.com/chhylp123/hifiasm
HiGlass	1.11.6	https://github.com/higlass/higlass
Merqury	MerquryFK	https://github.com/thegenemyers/MERQURY.FK
MitoHiFi	2	https://github.com/marcelauliano/MitoHiFi
PretextView	0.2	https://github.com/wtsi-hpag/PretextView
purge_dups	1.2.3	https://github.com/dfguan/purge_dups
sanger-tol/genomenote	v1.0	https://github.com/sanger-tol/genomenote
sanger-tol/readmapping	1.1.0	https://github.com/sanger-tol/readmapping/tree/1.1.0
YaHS	yahs-1.1.91eebc2	https://github.com/c-zhou/yahs

### Wellcome Sanger Institute – Legal and Governance

The materials that have contributed to this genome note have been supplied by a Darwin Tree of Life Partner. The submission of materials by a Darwin Tree of Life Partner is subject to the **‘Darwin Tree of Life Project Sampling Code of Practice’**, which can be found in full on the Darwin Tree of Life website here. By agreeing with and signing up to the Sampling Code of Practice, the Darwin Tree of Life Partner agrees they will meet the legal and ethical requirements and standards set out within this document in respect of all samples acquired for, and supplied to, the Darwin Tree of Life Project. 

Further, the Wellcome Sanger Institute employs a process whereby due diligence is carried out proportionate to the nature of the materials themselves, and the circumstances under which they have been/are to be collected and provided for use. The purpose of this is to address and mitigate any potential legal and/or ethical implications of receipt and use of the materials as part of the research project, and to ensure that in doing so we align with best practice wherever possible. The overarching areas of consideration are:

•   Ethical review of provenance and sourcing of the material

•   Legality of collection, transfer and use (national and international) 

Each transfer of samples is further undertaken according to a Research Collaboration Agreement or Material Transfer Agreement entered into by the Darwin Tree of Life Partner, Genome Research Limited (operating as the Wellcome Sanger Institute), and in some circumstances other Darwin Tree of Life collaborators.

## Data Availability

European Nucleotide Archive: *Coleophora flavipennella* (tipped oak case-bearer). Accession number PRJEB56048; https://identifiers.org/ena.embl/PRJEB56048. ( Wellcome Sanger Institute, 2022) The genome sequence is released openly for reuse. The *Coleophora flavipennella* genome sequencing initiative is part of the Darwin Tree of Life (DToL) project. All raw sequence data and the assembly have been deposited in INSDC databases. The genome will be annotated using available RNA-Seq data and presented through the Ensembl pipeline at the European Bioinformatics Institute. Raw data and assembly accession identifiers are reported in Table 1.

## Author information

Members of the University of Oxford and Wytham Woods Genome Acquisition Lab are listed here: https://doi.org/10.5281/zenodo.4789928.

Members of the Darwin Tree of Life Barcoding collective are listed here: https://doi.org/10.5281/zenodo.4893703.

Members of the Wellcome Sanger Institute Tree of Life programme are listed here: https://doi.org/10.5281/zenodo.4783585.

Members of Wellcome Sanger Institute Scientific Operations: DNA Pipelines collective are listed here: https://doi.org/10.5281/zenodo.4790455.

Members of the Tree of Life Core Informatics collective are listed here: https://doi.org/10.5281/zenodo.5013541.

Members of the Darwin Tree of Life Consortium are listed here: https://doi.org/10.5281/zenodo.4783558.
