# Supplementary material for: The genome sequence of the Tipped Oak Case-bearer, Coleophora flavipennella (Duponchel 1843)
Source: Wellcome Open Res. 2024 Jan 3;9:3. [Version 1] doi: 10.12688/wellcomeopenres.19917.1 (PMC11056683; doi:10.12688/wellcomeopenres.19917.1)
Supplement: Peer review history

# Reviewer report: Reviewer response for version 1

**DOI:** 10.21956/wellcomeopenres.22054.r77448

**Published:** 2024-05-22

**Authors:** Boyd A Mori

**Affiliations:** 1 University of Alberta, Edmonton, Alberta, Canada

This data note provides the first genome assembly for the Coleophora flavipennela. This is adding to the growing list of Coleophoridae sequenced by the Wellcome Sanger Institute. The rationale for sequencing the genome is clear, although perhaps a bit of a stretch without further background information on different birds and the ways they interact with coleophorids.

 Methods are spare, but clear and use the latest technology.

Are sufficient details of methods and materials provided to allow replication by others?

Yes

Is the rationale for creating the dataset(s) clearly described?

Partly

Are the datasets clearly presented in a useable and accessible format?

Yes

Are the protocols appropriate and is the work technically sound?

Yes

Reviewer Expertise:

entomology, genomics

I confirm that I have read this submission and believe that I have an appropriate level of expertise to confirm that it is of an acceptable scientific standard.

# Reviewer report: Reviewer response for version 1

**DOI:** 10.21956/wellcomeopenres.22054.r77446

**Published:** 2024-05-08

**Authors:** Panagiotis Ioannidis

**Affiliations:** 1 Foundation for Research & Technology Hellas, Crete, Greece

This manuscript describes the sequencing and assembly of a lepidopteran insect. The authors provide adequate background about the biology and the importance of this insect. The genome assembly is very good, judging from the provided assembly metrics. I only have a few minor comments:

 1) the authors report that the number of chromosomes is 55+1. Is this expected for the particular clade of Lepidoptera in which *C. flavipennella* belongs?

 2) in the first paragraph of the Methods section, it is mentioned that different individuals were used for the HiFi and HiC sequencing. I'm assuming that there is a reason the authors didn't use the same individual for both (small size perhaps?), but could they comment on it (and maybe add a sentence in the text)?

 3) Were the RNAseq data to which the authors are referring in the Data Availability section, generated within the frame of this manuscript? Because I couldn't see any information about transcriptome sequencing in the text of the manuscript.

 4) I think that there is a typo in the last paragraph of the Background section: "...but is likely to be being impacted..." should be "...but is likely to being impacted...".

Are sufficient details of methods and materials provided to allow replication by others?

Yes

Is the rationale for creating the dataset(s) clearly described?

Yes

Are the datasets clearly presented in a useable and accessible format?

Yes

Are the protocols appropriate and is the work technically sound?

Yes

Reviewer Expertise:

Insect genomics and transcriptomics

I confirm that I have read this submission and believe that I have an appropriate level of expertise to confirm that it is of an acceptable scientific standard.

# Reviewer report: Reviewer response for version 1

**DOI:** 10.21956/wellcomeopenres.22054.r77447

**Published:** 2024-04-28

**Authors:** Jerome Hui

**Affiliations:** 1 The Chinese University of Hong Kong, Hong Kong, Hong Kong

Boyes, Blaxter, and colleagues report the genome sequence of moth *Coleophora flavipennella* **(Duponchel, 1843). According to the Butterfly Conservation's Microlepidoptera Report (No. S12-02) in 2012, this species was classified as common in the UK. Molecular data of this species are scarce prior to this report, and are mainly confined to COI sequences (and one nuclear gene) deposited to the NCBI database. This new genome resource will be very useful for further studies, such as understanding their ecology, population structure, response to environment/climate changes, and evolutionary relationships with other lepidopterans.

 This genome resource is excellent from the summary statistics, with high BUSCO numbers, high sequence continuity (scaffold N50), and majority of sequences contained on the 55 pseudochromosomes (plus 2 sex chromosomes and mitochondrion). To sum up, this is another valuable contribution from the Darwin Tree of Life.

Are sufficient details of methods and materials provided to allow replication by others?

Yes

Is the rationale for creating the dataset(s) clearly described?

Yes

Are the datasets clearly presented in a useable and accessible format?

Yes

Are the protocols appropriate and is the work technically sound?

Yes

Reviewer Expertise:

Genomics, evolution, invertebrates

I confirm that I have read this submission and believe that I have an appropriate level of expertise to confirm that it is of an acceptable scientific standard.

# Reviewer report: Reviewer response for version 1

**DOI:** 10.21956/wellcomeopenres.22054.r74474

**Published:** 2024-04-28

**Authors:** Paula Escuer

**Affiliations:** 1 University of Neuchâtel, Neuchâtel, Switzerland

The authors presented the chromosome level gnome sequence of a female individual of *Coleophora flavipennella* (The Tipped Oak Case-bearer). The final assembly presents a total length of 989.3 Mb in 184 scaffolds, including the W and Z sex chromosomes. The N50 is 17.9 Mb and 99.38% of the assembly is condensed in 57 chromosomal-level scaffolds. It is also included the alternative haplotype and mitochondrial genome. The BUSCO pipeline results showed a good quality and contiguity of the genome, with 97.9% of complete genes found. This high quality assembly is an important contribution that will allow understand the genomics and evolution of the Lepidoptera order.

Are sufficient details of methods and materials provided to allow replication by others?

Yes

Is the rationale for creating the dataset(s) clearly described?

Yes

Are the datasets clearly presented in a useable and accessible format?

Yes

Are the protocols appropriate and is the work technically sound?

Yes

Reviewer Expertise:

Genomics, Evolution, Biodiversity, Speciation

I confirm that I have read this submission and believe that I have an appropriate level of expertise to confirm that it is of an acceptable scientific standard.

# Reviewer report: Reviewer response for version 1

**DOI:** 10.21956/wellcomeopenres.22054.r73319

**Published:** 2024-02-26

**Authors:** Li-Wei Wu

**Affiliations:** 1 Tunghai University, Taichung, Taichung City, Taiwan

This report clearly presents the genomic information of *Coleophora flavipennella*, including the NGS technologies employed and the analytical methods used. While it doesn't delve deeply into the practical applications or the implications for Lepidoptera research, the content meets the immediate approval requirements. Moreover, the data provided will be advantageous for researches into the evolution of Lepidoptera insects or for studies on pest control.

 Here are some minor suggestions to improve the content for reader:

 1.      A citation should be provided for the use of BUSCO in the methodology section.

 2.      The mitogenomic sequence should give a GenBank accession number to increase the visibility and usability.

Are sufficient details of methods and materials provided to allow replication by others?

Yes

Is the rationale for creating the dataset(s) clearly described?

Yes

Are the datasets clearly presented in a useable and accessible format?

Yes

Are the protocols appropriate and is the work technically sound?

Yes

Reviewer Expertise:

Population genetics; Historical biogeography; development of genome-wide SNP

I confirm that I have read this submission and believe that I have an appropriate level of expertise to confirm that it is of an acceptable scientific standard.
